# Lyse des vertèbres dorsales révélant un anévrysme chronique rompu contenu: à propos d’un cas

**DOI:** 10.11604/pamj.2019.32.32.17851

**Published:** 2019-01-17

**Authors:** Diekouadio Fabrice, Bellamlih Habib, Laamrani Fatim Zahra

**Affiliations:** 1 Service d’Imagerie Médicale, CHU Ibn Sina, Faculté de Médecine et de Pharmacie Mohamed V, Rabat, Maroc

**Keywords:** Anévrisme chronique rompu contenu, lyse, vertèbres, Chronic contained rupture of an aneurysm, lysis, vertebral body

## Abstract

Les anévrismes chroniques rompus contenus représentent une entité extrêmement rare, qui peut faire suite à une rupture d'anévrisme. En effet, la complication la plus à craindre de l'anévrisme est la rupture, qui est souvent mortelle. Toutefois exceptionnellement, il se formera un hématome suite à la rupture, lequel sera contenu par les structures anatomiques avoisinantes; ce qui permettra en outre d'arrêter la fuite. Contrairement à la rupture classique, les signes de choc hémorragique seront absents; remplacés par une simple douleur modérée, et des signes souvent atypiques tels que la lyse du rachis dorsal par l'hématome constitué, découverte à l'imagerie dans notre cas. La connaissance de cette entité est importante, car la rupture chronique nécessite une prise en charge chirurgicale; du fait du risque de rupture ultérieure de l'hématome.

## Abstract (en)

Chronic contained ruptures of aneurysms are extremely rare. They can result from aneurysm rupture. Indeed, the most dreaded complication of aneurysm is its rupture, which is often fatal. However, haematoma can exceptionally occur after rupture, which is contained by the surrounding anatomical structures, stopping aneurysm from leaking. Unlike the classic rupture, there are no signs of hemorrhagic shock. They are replaced by moderate pain and, often, by atypical signs such as lysis of the dorsal vertebral bodies caused by the occurrence of an hematoma detected by imaging, as in our case. It’s important to know this disorder because chronic rupture requires surgical management due to the risk of later rupture of the haematoma.

## Introduction

Un anévrisme est une dilatation localisée de la paroi d'une artère avec perte du parallélisme des parois, aboutissant à la formation d'une poche de taille variable, communiquant avec l'artère au moyen du collet. Sa forme habituelle est celle d'un sac, son diamètre pouvant atteindre plusieurs centimètres. La complication la plus grave est la rupture d'anévrisme; qui peut engager le pronostic vital. Dans de rares cas, cette rupture se retrouve contenue par les structures anatomiques adjacentes : on parle alors d'anévrysme rompu contenu.

## Patient et observation

**Figure 1:**
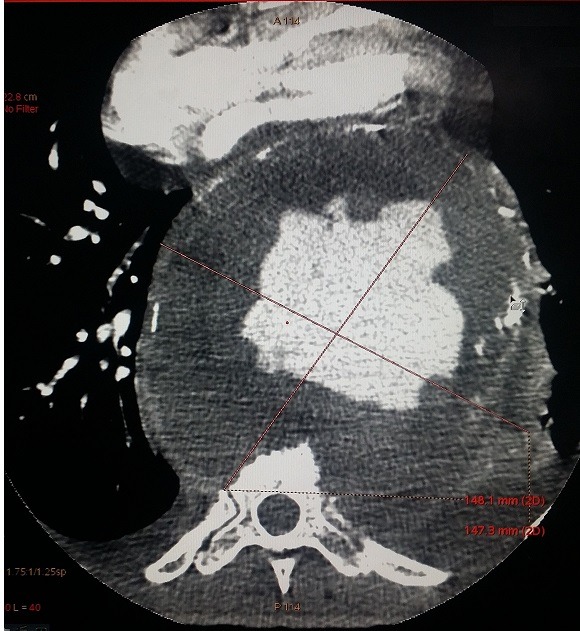
Coupe axiale montrant un volumineux anévrysme de l’aorte thoracique descendante; refoulant en avant les cavités cardiaques gauches

**Figure 2:**
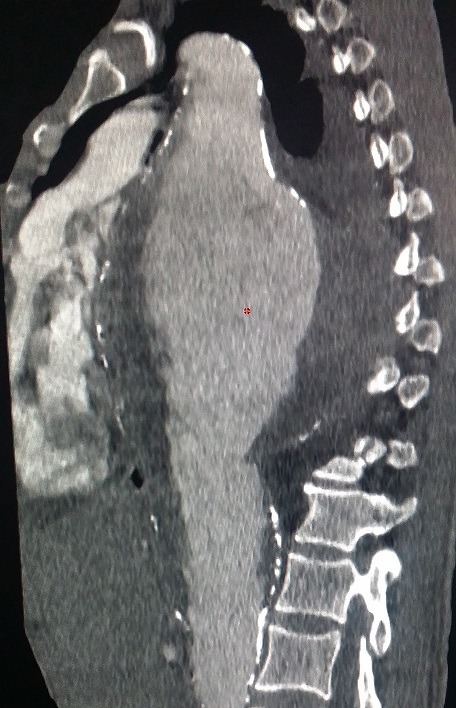
Coupe sagittale montrant une discontinuité des calcifications pariétales de la paroi postérieure de l’aorte thoracique descendante

**Figure 3:**
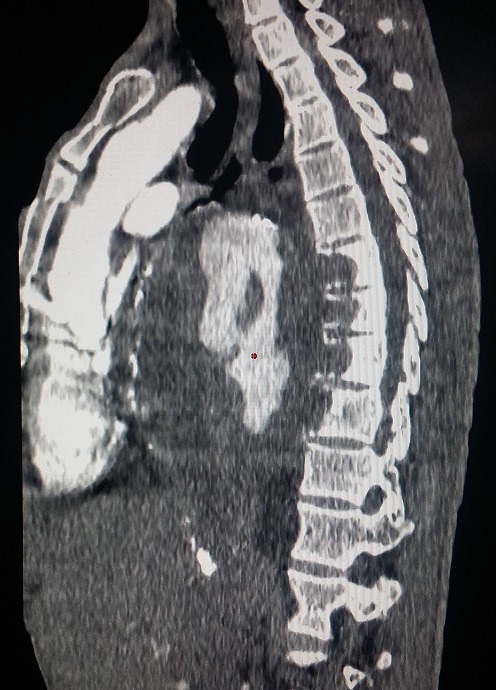
Coupe sagittale montrant une lyse des corps vertébraux de D7 à D10 par l’hématome

**Figure 4:**
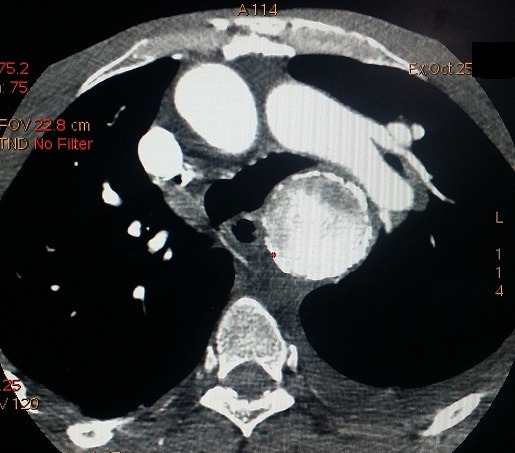
Coupe axiale montrant un effet de masse sur l’artère pulmonaire gauche, qui reste perméable

Nous rapportons le cas d'un patient de 69 ans, tabagique chronique non sevré; suivi pour maladie de Leo Buerger, connu porteur d'un anévrysme thoracique mal suivi. Cette tétraparésie s'accompagne d'une dyspnée stade II de la NYHA; avec des douleurs thoraciques modérées. À l'admission, la tension artérielle était à (142-84 mm Hg). Il a bénéficié d'une radiographie thoracique; qui a mis en évidence une masse médiastinale postérieure. Devant la suspicion d'une rupture d'anévrysme thoracique, un angioscanner thoracique a été réalisé, objectivant: 1) un volumineux anévrysme de l'aorte thoracique descendante et de l'aorte abdominale, mesurant 148x148mm (Figure 1); 2) cet anévrysme partiellement thrombosé est le siège de calcifications pariétales discontinues, témoignant d'une fissuration contenue (Figure 2); 3) l'hématome péri-anévrysmal englobe les corps vertébraux de D7 à D10, dont il lyse les corticales antérieures (Figure 3); a) en avant: comprime les cavités cardiaques gauches (Figure 1); b) en haut: écarte l'artère pulmonaire, sans signes de thrombose évidente (Figure 4); c) un épanchement pleural bilatéral de faible abondance, plus marqué à gauche (Figure 4).

## Discussion

Les anévrismes peuvent toucher n'importe quel vaisseau sanguin, notamment le polygone de Willis, l'aorte thoracique et l'aorte abdominale. Lorsque l'anévrisme augmente en taille, le risque de rupture augmente [1]. La rupture d'anévrysme est une complication redoutable [2]. Elle s'accompagne d'un état de choc hémorragique avec tachycardie, hypotension artérielle. La particularité des anévrysmes chroniques contenus réside dans le fait qu'il existe une différence notable avec les ruptures classiques, sur le plan clinique. En effet, l'état hémodynamique reste globalement stable, ce qui peut entrainer un retard diagnostic. Les symptômes sont donc liés à la localisation de l'anévrysme. Dans notre cas, la tétraplégie s'explique par l'érosion des corps vertébraux de D7 à D10, par l'hématome, éventuellement d'une compression médullaire; la dyspnée par le caractère compressif de l'anévrysme, et la turbulence du flux sanguin. La rupture aortique chronique contenue est une pathologie extrêmement rare: seulement 138 cas ont été rapportés dans la littérature à ce jour [3]. La rupture chronique contenue se distingue des anévrysmes non rompus par une solution de continuité de la paroi; il se crée un hématome par rupture de la paroi artérielle, mais qui est contenu et confiné à côté du vaisseau par le tissu environnant. Cette cavité remplie de sang finit par thromboser afin d'arrêter la fuite, mais risque par la suite de se rompre hors des tissus environnants [4]. La TDM est l'examen de choix pour établir le diagnostic, surtout en cas de lyse vertébrale par l'hématome. On peut parfois retrouver une discontinuité de calcifications pariétales au niveau de l'hématome. Bien que contenus, ces anévrysmes doivent être l'objet d'une prise en charge chirurgicale [5].

## Conclusion

Les anévrismes chroniques rompus contenus représentent une évolution exceptionnelle de la rupture d'anévrisme classique. Leurs manifestations cliniques sont moins bruyantes; la TDM est nécessaire pour confirmer le diagnostic. Une prise en charge chirurgicale doit être programmée pour rétablir la continuité de la paroi.

## Conflits d’intérêts

Les auteurs ne déclarent aucun conflit d'intérêts.
